# Small but Challenging Conjunctival Melanoma: New Insights, Paradigms and Future Perspectives

**DOI:** 10.3390/cancers13225691

**Published:** 2021-11-14

**Authors:** Sacha Nahon-Estève, Corine Bertolotto, Alexandra Picard-Gauci, Lauris Gastaud, Stéphanie Baillif, Paul Hofman, Anaïs Groulier, Célia Maschi, Jean-Pierre Caujolle, Sandra Lassalle, Arnaud Martel

**Affiliations:** 1 Ophthalmology Department, Centre Hospitalier Universitaire de Nice, Université Côte d’Azur, 06000 Nice, France; baillif.s@chu-nice.fr (S.B.); maschi.c@chu-nice.fr (C.M.); jeanpierre.caujolle@neuf.fr (J.-P.C.); martel.a@chu-nice.fr (A.M.); 2 Department of Biology and Pathologies of Melanocytes, Team1, Equipe Labellisée Ligue 2020 and Equipe Labellisée ARC 2019, Centre Méditerranéen de Médecine Moléculaire, INSERM, 06200 Nice, France; Corine.Bertolotto@unice.fr; 3 Dermatology Department, Centre Hospitalier Universitaire de Nice, Université Côte d’Azur, 06000 Nice, France; picard-gauci.a@chu-nice.fr; 4 Antoine Lacassagne Cancer Centre, Oncology Department, Université Côte d’Azur, 06000 Nice, France; lauris.GASTAUD@nice.unicancer.fr; 5 FHU OncoAge, Institute for Research on Cancer and Aging, Nice (IRCAN), Université Côte d’Azur, 06000 Nice, France; hofman.p@chu-nice.fr (P.H.); lassalle.s@chu-nice.fr (S.L.); 6 Biobank BB-0033-00025, Laboratory of Clinical and Experimental Pathology, Centre Hospitalier Universitaire de Nice, Université Côte d’Azur, 06000 Nice, France; 7 Antoine Lacassagne Cancer Center, Department of Radiation Oncology, Université Côte d’Azur, 06000 Nice, France; Anais.GROULIER@nice.unicancer.fr

**Keywords:** conjunctival melanoma, tumour recurrence, metastases, orbital exenteration, targeted therapies, immunotherapy, proton beam radiotherapy

## Abstract

**Simple Summary:**

Conjunctival melanoma (CM) is a small but highly aggressive and infiltrative periocular malignancy. Despite wide surgical excision followed by adjuvant therapy, about one third and one quarter of patients will experience local recurrence and metastatic spread, respectively. The management of locally advanced (≥T2) tumours may require mutilating surgeries such as orbital exenteration to achieve local control. The last decade has been marked by the emergence of eye-sparing strategies based on wide surgical excision followed by adjuvant proton beam therapy. More recently, new genetic and immunological insights have incriminated several signalling pathways (MAPK, PI3K-AKT) and immune cells, making CM a “targetable” malignancy. Anti-BRAF and anti-MEK targeted therapies and immunotherapies have revolutionized the current management of CM through the use of new eye-sparing strategies and treatment of metastases.

**Abstract:**

Although its incidence has increased over the last decades, conjunctival melanoma (CM) remains a rare but challenging periocular malignancy. While there is currently no recognized standard of care, “no-touch” surgical excision followed by adjuvant treatments is usually recommended. Despite its small size, managing CM is challenging for clinicians. The first challenge is the high risk of tumour local recurrence that occurs in about one third of the patients. The management of locally advanced CM (≥T2) or multiple recurrences may require mutilating surgeries such as orbital exenteration (OE). The second challenge is the metastatic spread of CM that occurs in about one quarter of patients, regardless of whether complete surgical excision is performed or not. This highlights the infiltrative and highly aggressive behaviour of CM. Recently, attention has been directed towards the use of eye-sparing strategies to avoid OE. Initially, wide conservative surgeries followed by customized brachytherapy or radiotherapy have appeared as viable strategies. Nowadays, new biological insights into CM have revealed similarities with cutaneous melanoma. These new findings have allowed clinicians to reconsider the management of locally advanced CM with “medical” eye-sparing treatment as well as the management of metastatic spread. The aim of this review was to summarize the current and future perspectives of treatment for CM based on recent biological findings.

## 1. Introduction

**Figure 1:**
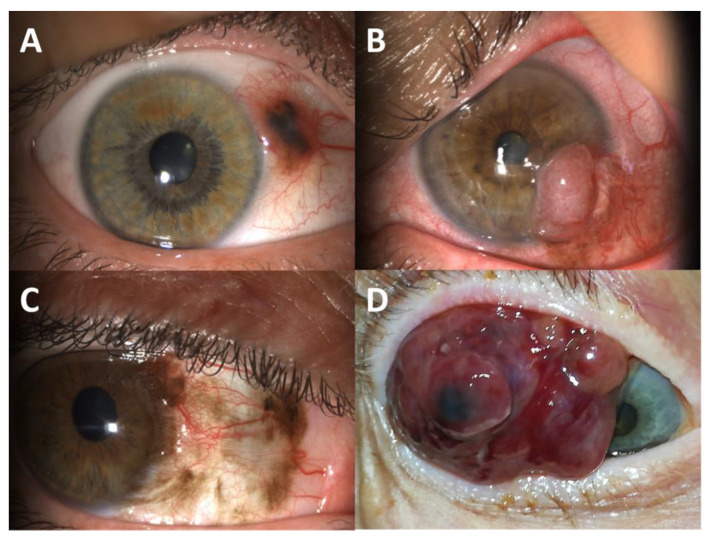
Clinical aspect of conjunctival melanoma (CM) upon presentation or after recurrence: (**A**) CM arising from a naevus; (**B**) Recurrence of achromic CM with primary acquired melanosis (PAM) (first surgery performed in another area without “no-touch” surgery); (**C**) Extended multifocal CM arising from PAM; (**D**) Early and massive recurrence of CM despite wide local excision followed by proton beam radiotherapy. The patient underwent orbital exenteration and died from metastatic spread of his CM thereafter.

Despite their small size, various types of melanomas may be found in the eye and periocular area. The generic term “eye melanoma” encompasses several radically opposed entities such as conjunctival melanoma (CM), uveal melanoma, cutaneous melanoma, primary orbital melanoma and melanoma metastasis. CM is a rare periocular malignancy that still represents a challenge for clinicians despite its small size (Figure 1) [1]. CM is an aggressive, infiltrative and radioresistant malignancy with a propensity for recurrence and spreading (Figure 1D) [2,3,4]. To date, there is no standard of care for CM and this could be explained by its scarcity and the lack of high-quality studies. Despite complete surgical excision, local recurrence and metastatic spread occur in about one third and one quarter of patients, respectively [5,6,7]. In some cases, radical and disfiguring surgeries such as orbital exenteration (OE) are needed to achieve tumour control [8]. Nevertheless, OE has failed to improve the overall survival. Therefore, new “eye-sparing” strategies based on wide local surgical excision followed by personalized proton beam radiotherapy (PBRT) have progressively emerged as viable strategies, even in locally advanced CM. Despite being anatomically close, CM and uveal melanoma are very different genetically [9,10]. CM shares similarities with both cutaneous melanoma and mucosal melanoma, including their infiltrative nature, their lymphatic and hematogenous spread and the presence of *BRAF*, *NRAS*, *NF1* and *Kit* mutations [11,12]. Therefore, like cutaneous melanoma, CM has become a “targetable” malignancy [1]. The efficacy of BRAF and MEK inhibitors as well as immune checkpoint inhibitors (anti-CTLA4, anti-PD1, anti-PDL1) has been shown in recurrent, locally advanced and/or metastatic CM. Although the literature is scarce, a new treatment paradigm towards the use of less invasive surgeries and treatment personalization seems to have emerged. The aim of this review was to summarize the current management of CM based on recent genetic findings and to focus on the advent of new eye-sparing strategies.

## 2. Method for Literature Search

A thorough literature search was performed on Medline over the 2001–2021 period using the main search term “conjunctival melanoma” and the following terms “biology”, “genetic”, “immunology”, “tumour recurrence”, “survival”, “orbital exenteration”, “eye-sparing surgery”, “targeted therapy”, and “immunotherapy”. Titles and abstracts were reviewed by two independent authors (S.N.-E., A.M.). References were also identified from citations in papers identified in the original search. One hundred and eighty-eight original articles, case reports and reviews focused on the recent biological findings and management strategies of CM written in English or in French were considered and 125 were selected.

## 3. Biology of Conjunctival Melanoma

**Figure 2:**
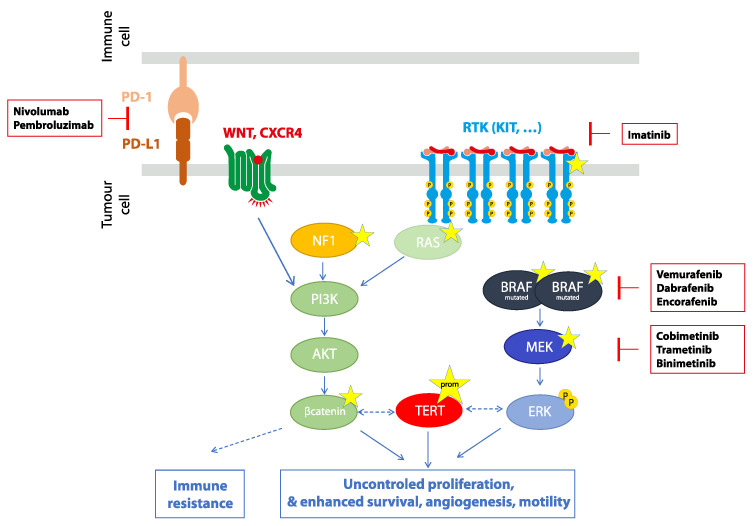
Biological pathways and mutations involved in melanogenesis. Mutations are indicated by yellow stars.

Although being anatomically close to the uveal tract, CM is genetically close to cutaneous and mucosal melanomas. Several mutations have been shown to disturb several signalling pathways such as the ‘MAPK’ (mitogen-activated protein kinase, also known as ‘RAS-RAF-MEK-ERK’) pathway and the ‘PI3K-AKT’ (also known as ‘PI3K-AKT-mTOR’) pathway, as well as their regulators, such as NF1 or receptor tyrosine kinases such as KIT [12]. With *TERT* promoter mutations, all these abnormalities contribute to the genetic landscape of CM (Figure 2).

Recent decades have been marked by better knowledge of CM oncogenesis. First, the increasing incidence of CM over time, especially in men, and the identification of UV signatures in CM with differences between bulbar and tarsal lesions, support a role of UV in CM development [13,14,15,16,17,18,19,20,21]. Like cutaneous melanomas, it has been proposed to classify CMs according to their mutational status, resulting in groups of *BRAF*-mutated, *NRAS*-mutated, *NF1*-mutated and triple-wild type (WT) melanomas [11,22]. A number of these mutations are known to be related to a chronic sun exposure, whereas the triple-WT group is not [21,23]. Taken together, these data support the fact that CMs are a biologically distinct, heterogeneous group of melanomas with a mixed phenotype and features of mucosal melanomas associated with DNA damage induced by chronic UV exposure. Second, even if a clear association between the clinical features or prognosis and the genetic abnormalities have not been established, several results suggest that some mutations could be associated with specific tumorigenesis pathways for some CM subgroups [12,21]. *CTNNB1* mutations are more common in nevi-derived CMs, suggesting a pivotal role of the Wnt pathway in their tumorigenesis, whereas the presence of *KIT*/*SF3B1* mutations suggests a mucosal-specific tumorigenic pathway as for other mucosal melanomas [11,24,25,26,27]. Of note, in cutaneous melanomas, β-catenin has been associated with an immune resistance [28,29]. Finally, the study of the local tumour microenvironment (TME) allows a better understanding of the mechanisms of CM tumorigenesis. The TME includes the surrounding immune cells (both innate and adaptative immune cells), vascular endothelial cells, extracellular matrix proteins, fibroblasts, and signalling molecules. Several studies have shown that an outgrowth of lymphatic vessels is concomitant with the development of CM, possibly due to the increased expression of lymphangiogenic and chemotactic factors at the invasive edge of CM [30,31,32]. This mechanism could also explain the migration to and invasion of CM cells into the lymphatic vessels [33,34]. This prolymphangiogenic potential does not seem to be only associated with CM cells, but could also involve cells present in the TME [35]. Regarding innate and adaptive immune cells, no clear conclusion can be drawn because of the contradictory results regarding the role of tumour infiltrate lymphocytes (TILs) and tumour-associated macrophages (TAMs) [36,37,38,39,40,41]. Elevated levels of HLA Class I are associated with more TILs and TAMs and the expression of PD-L1 in CM seems almost similar to that found in cutaneous melanoma, even if lower percentages of expression levels have been reported [41,42,43]. The relevance of this predictive effect may be limited because, as with cutaneous melanoma, CMs with high PD-L1 levels have been shown to respond to PD-1 inhibitors [44].

## 4. Conventional Treatment for Conjunctival Melanoma

Despite great advances in the understanding of CM biology, CM treatment has not fundamentally evolved and there is still no clear consensus on the optimal adjuvant treatment to be given after local excision due to a lack of prospective randomized controlled trials.

### 4.1. Surgery

“No-touch surgery” is the only technique that is widely recognized as the gold standard, but the surgical instruments and surgeon’s gloves mandatorily need to be regularly changed to prevent tumour cell seeding outside the surgical site [45]. This surgery consists of removing the tumour with clear macroscopic margins without touching the tumour, and may be combined with absolute alcohol corneal epitheliectomy in the case of corneal involvement. Except in special cases, incisional biopsy should not be performed. However, there is no consensus on the target surgical “tumour-free” margin (range: 2–5 mm) [45,46]. General anaesthesia is mandatory, since local anaesthetic injection is known to disrupt the tumour architecture and to promote local dissemination. Direct conjunctival closure is recommended whenever possible using clean instruments. If direct closure is not possible, amniotic membrane graft may be used to close the surgical wounds [47,48,49,50]. The initial surgery is an important part of CM management, although this is not systematically reported in studies. Interestingly, trends toward higher local recurrence rates have been reported in patients managed outside tertiary cancer centres [5,51]. In daily practice, most ocular oncology centres have to manage patients who have previously been operated on in other settings without respecting the aforementioned surgical rules. This partly contributes to the disparate results reported across different centres worldwide, depending on the percentage of patients primarily managed in these centres [5,51,52,53]. For example, Thariat et al. have reported a 5-year local recurrence rate of 33.2% (20.8%; 46.1%) for all patients treated at the cyclotron of Nice, whereas this rate decreased to 24.3% (8.5%; 44.5%) when the first surgery was directly performed by an ocular oncologist [5].

Another debate regarding surgery is the benefit of performing a sentinel lymph node (SLN) biopsy. The SLN is the first lymph node(s) to be invaded by tumour cells during lymphatic metastatic spread. SLN biopsies may help to initiate early treatment before the appearance of systemic metastases. They may also allow identifying patients with subclinical nodal metastases that would be missed on clinical or ultrasound examination alone. In case of CM, tumour-positive SLNs have been identified in 10 out of 85 patients (11.8%) with a thickness of 3.1–8 mm in 6 out of the 10 patients for whom these data were reported [54]. SLN biopsies are proposed when the CM thickness is greater than 2 mm or ranges between 1 and 2 mm, or when tumour ulceration is present but the impact of such procedures on patient outcomes remains to be confirmed [6,55,56]. The benefits of these procedures in terms of recurrence-free survival and overall survival must be balanced with the procedure morbidity [56,57,58]. Moreover, up to 26% of patients were found to have distant metastatic disease without local lymph-node involvement [59]. Currently, routine staging consists of general physical and ophthalmic examination, completed by a baseline imaging study for detecting regional lymph nodes and distant metastases. Regional lymph node ultrasounds, liver computed tomography (CT), magnetic resonance imaging (MRI) or ultrasound, brain MRI, and chest CT or positron emission tomography–CT scanning should be considered, although there is no clear recommendation on what imaging to do and when [6,60,61].

### 4.2. Adjuvant Therapies

As shown in Table 1, the ideal adjuvant therapy is debated and no standard of care has been proposed. Adjuvant therapy mainly depends on the local ocular oncology centre practice. Many authors recommend performing intraoperative cryotherapy at the surgical margins, while others have abandoned this technique and prefer to use adjuvant topical chemotherapy, brachytherapy or PBRT [5,45,51,62,63,64,65,66]. CM is a rare tumour and no studies have compared the different adjuvant treatments. Another major concern is the time interval between the initial surgery and the administration of adjuvant treatment. It has been shown that the longer the time interval, the higher the risk of local recurrence [67,68]. Several factors should be taken into account, such as the tumour origin (i.e., PAM versus naevus), location (i.e., bulbar versus tarsal) and extent, and the associated histologic prognostic factors.

#### 4.2.1. Cryotherapy

Cryotherapy is used intraoperatively and a double freeze–thaw cycle is applied, during which the cryoprobe remains in place for 10–20 s until an ice ball forms without touching the scleral bed to avoid any risk of damage to the underlying tissue (retinal or ciliary body damage/burn, uveitis, cataract formation) [64]. Although earlier reports have suggested that a temperature between −15 °C and −20 °C was adequate for treating intraepithelial growth, recent data indicate that applying a double freeze–thaw cycle at a temperature between −70 °C and −80 °C is more appropriate because it is not associated with major local adverse effects [2,64]. In 1993, De Potter et al. have shown significantly higher CM local recurrence rates after surgical treatment alone (68%) rather than after surgery combined with cryotherapy (18%) [66]. Since then, cryotherapy has been proposed as an adjuvant therapy after excisional biopsy. However, Jakobiec et al. have failed to show any benefit of cryotherapy on the prevention of metastatic disease [64].

#### 4.2.2. Topical Therapy

Topical chemotherapy is another adjuvant therapy that may be considered when the surgical margins of CM show PAM with atypia or residual intraepithelial disease postoperatively [76,77]. Repeated surgical excision is mandatory in case of surgical margins invaded by CM. Topical mitomycin C (MMC) 0.04% is the most commonly used chemotherapy since its first attempt by Finger et al. [46]. Topical chemotherapy allows the treatment of intraepithelial cancer cells throughout the conjunctiva. MMC is preferred to 5-fluorouracil (5-FU). Unlike 5-FU that only acts at the S phase of the cell cycle, MMC targets all of the phases of the cell cycle and induces a scission of the tumour DNA that persists even after treatment discontinuation [78,79]. Complications, such as kerato-conjunctivitis and epiphora, have been reported with MMC [80].

When MMC is poorly tolerated, topical interferon alpha 2b (IFN-α2b) may be a viable alternative with a better tolerance profile. Several studies have shown that CMs carry Interferon receptors. Therefore, IFN-α2b may act directly via a cytotoxic mechanism [81]. In addition, IFN-α2b may act via an indirect mechanism by upregulating the expression of HLA Class I, thereby enhancing the activity of cytotoxic CD8+ T cells, natural killer cells, and macrophages [12]. The efficacy of IFN-α2b as an adjuvant therapy is difficult to assess because the data are limited. Many case series have shown that adjuvant IFN-α2b induced a long-term remission, even in cases with residual PAM with atypia [82].

#### 4.2.3. Adjuvant Radiotherapy

Several ocular oncology centres use radiotherapy (brachytherapy or external beam radiotherapy (EBRT)) as an adjuvant treatment for invasive CM. Brachytherapy is widely used as an adjuvant radiotherapy for T1 and T2 CM [6,52,69,70,71,74,75]. Iodine-125, Strontium-90 or Ruthenium-106 have been used. The choice of treatment with Strontium-90 or Ruthenium-106 is usually based on the experience of the clinician and the availability of the materials. While Strontium-90 is applied in an outpatient setting with multiple short fractions, Ruthenium-106 is applied in one continuous setting, and often requires an overnight stay at the hospital. Four case series, including a total of 97 patients, have studied brachytherapy as an adjuvant therapy after surgical excision. Overall, 13 out of the 97 (13.4%) cases experienced local recurrences and 2 (2.1%) cases developed new tumours in other areas due to PAM [52,71,72]. One study has reported favourable results after a single-fraction adjuvant electronic brachytherapy session for 5 cases of early CMs (one with PAM) with a mean follow-up of 47.2 months (range: 31–60 months) [83]. No local relapse, no metastasis and no deaths were reported, but further studies are needed to confirm the place of single-fraction adjuvant electronic brachytherapy.

**Figure 3:**
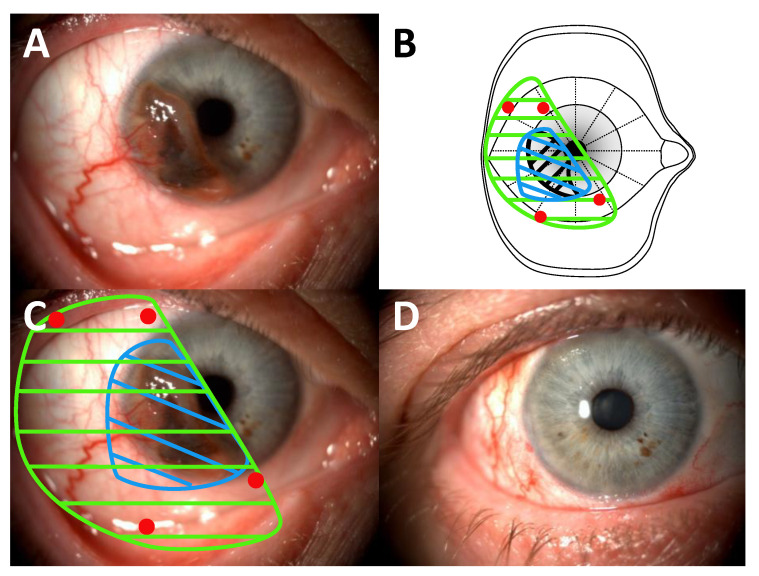
Adjuvant proton beam irradiation plan for conjunctival melanoma (CM). (**A**) CM before surgical resection; (**B**) Drawing of the treatment plan on the tantalum clip positioning diagram; (**C**) Simulation of the treatment plan on the preoperative primary position photography; (**D**) Clinical aspect 1 year after treatment.

PBRT is a type of external irradiation therapy used for small and locally advanced CMs. As an alternative to brachytherapy and exenteration, PBRT may be used for T1, T2 and T3 stages. In our tertiary care centre, PBRT is applied over 2 weeks with a total dose of 45 Gy, including 31.2 Gy delivered in the main field and 13.8 additional Gy as a “boost” in high-risk areas (Figure 3). With this technique, Thariat et al. have reported a 5-year rate of local recurrence of 33.2% in 92 patients [5]. More interestingly, when the first surgery was performed by a specialized ocular oncologist (42 patients), only 24.3% of patients experienced a local failure at 5 years. The patterns of local relapse for all patients were in-field in 16.0% of cases (*n* = 4), and marginal or out-of-field in 52.0% of cases (*n* = 14) but could not be assessed in 28.0% of patients (*n* = 7). Salvage OE was needed in 13 (14%) patients. In a case series of 89 patients with T2 and T3 CM, Scholz et al. have shown that OE was needed in 18% of patients at 5 years [62]. Compared to OE, adjuvant radiotherapy has the advantage to be associated with a 69% 5-year cumulative likelihood of eye preservation. PBRT is an effective conservative treatment for managing relapse in about half of cases [73]. In total, three case series, including a total of 201 patients, have investigated PBRT as an adjuvant therapy after surgical excision [5,62,73]. Overall, 60 out of the 201 (29.9%) patients experienced local recurrences, and 33 (16.4%) underwent secondary OE. Regardless of the radiotherapy modality chosen, the 5-year rates of melanoma-related metastases (5.5% and 6%) and melanoma-related mortality (3.6% and 3%) were similar [5,52].

## 5. Orbital Exenteration for Conjunctival Melanoma

In the case of locally advanced conjunctival melanoma (e.g., orbital invasion), eye consertion may be not possible. OE is a radical and disfiguring surgery consisting of the removal of all the orbital contents with a subperiosteal dissection [84]. OE is associated with a dramatic psychological impact. OE is rarely performed with less than one hundred procedures performed yearly in France, corresponding to an annual incidence of 0.13/100,000 inhabitants [85]. In tertiary ocular oncology centres, CM is often the leading cause of OE [5,8,86,87,88]. OE is rarely performed as a first-line treatment for CM and is usually recommended in patients with an orbital involvement or multiple tumour recurrence [5,8,55,89]. A recent study has recommended the performance of early OE (i.e., before the occurrence of 4 local recurrences) to reduce the risk of metastatic spread [89]. Tumour-related death has been associated with de novo origin, non-limbal location, large tumour size, orbital invasion, nodular growth, and multicentric origin.

**Figure 4:**
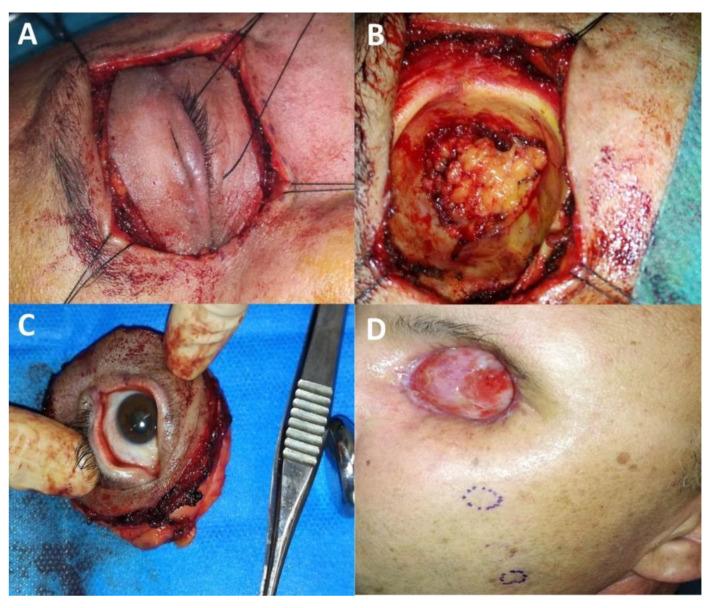
Orbital exenteration (OE) for recurrent tarsal conjunctival melanoma: (**A**) Total OE with incisions performed circumferentially to the orbital rim; (**B**,**C**) Orbital socket and specimen aspect; (**D**) Lymph node dissemination was palpable 3 months after surgery (purple circles). The orbital socket was reconstructed with an artificial dermis template followed by spontaneous epithelialization. Note the “bowl-shaped” aspect of the orbital socket.

Several surgical techniques have been described, including eyelid-sparing and total or extended OE (Figure 4) [90]. There is still no consensus on the best surgical technique for treating CM. Shields et al. have systematically performed eyelid-sparing OE. In our experience, we prefer to perform total OE [8,55]. The objective of OE is to remove the entire tumour with clear surgical margins. Obtaining clear surgical margins has been associated with fewer local recurrences and is thought by certain authors to reduce the risk of metastatic spread [8,91]. Therefore, wide surgical excision with clear surgical margins ≥ 5–10 mm is usually advocated [84]. In our opinion, it is not possible to obtain clear surgical margins ≥ 5–10 mm with eyelid-sparing OE given the close connection between the conjunctiva (bulbar and/or tarsal) and the eyelid. However, it should be noted that Shields et al. and Jayaprakasam et al. have reported low tumour recurrence rates after eyelid-sparing OE for anteriorly located malignancies [55,92].

Several orbital socket reconstruction techniques have been described, ranging from simple spontaneous granulation to highly complicated and time-consuming free flaps [84]. Extended OE (i.e., bony orbital removal) is exceptionally required when managing locally advanced CM. In our opinion, the first objective of the reconstructive surgery is to obtain a “bowl-shaped” rather than a “bulky-shaped” orbital socket to facilitate subsequent facial prosthesis delivery and the early diagnosis of tumour recurrence [84]. Bowl-shaped sockets are more easily obtained when spontaneous granulation, split-thickness skin graft, and frontalis or cheek flaps are performed (Figure 4D). Therefore, we recommend using these reconstructive techniques in the case of CM rather than free flaps. Recent lying materials, such as artificial dermis grafts, have been associated with favourable orbital socket healing results and reduced intraoperative morbidity [93]. Facial prostheses are better retained by osseointegrated orbital implants that can be placed during or after ablative surgery [94]. New technologies, including laser, CAD/CAM (Computer aided design/computer aided manufacturing) and 3D printing, have recently emerged as innovative tools to improve facial prosthesis delivery [95].

Despite all these recent surgical and technological progresses, about one third of the patients prefer to wear an eye patch rather than a facial prosthesis [75]. Although rare, several life-threatening complications, such as cerebrospinal fluid leakages, have been reported after OE [96]. In addition, recent controverses regarding the benefit/risk ratio of OE have emerged. Several studies have found a decreased rate of local recurrences after OE, but they have failed to show that OE could decrease the risk of metastatic spread (Figure 4D) [84]. The mean 1- and 5-year overall survival after OE ranges between 69.1% and 97% and between 37% and 92%, respectively. In their study in 95 CM patients, Paridaens et al. have failed to demonstrate the usefulness of OE to improve the overall survival [97]. They have advocated the use of wide local debulking surgery combined with adjuvant treatments including radiotherapy and to use OE as a palliative treatment. Finally, new targeted therapies and immunotherapies have recently emerged as a viable option for recurrent, locally advanced, or metastatic CM. Therefore, the use of OE has become increasingly questionable in CM management. The management decisions should be made by a multidisciplinary team and include new biological insights in order to personalize treatment.

Enucleation and evisceration are usually not recommended for the management of conjunctival melanoma. These surgeries require conjunctiva mobilization, which could promote local and regional dissemination. However, in certain highly selected cases, evisceration can be performed. For example, in the case of intractable eye pain, or scleral perforation following proton beam radiotherapy and if tumor control is achieved, eye evisceration can be performed. Care should be taken not to mobilize the conjunctiva and the sclera. Therefore, we do not recommend the insertion of an orbital implant, which requires scleral and conjunctiva mobilization to avoid further exposure. Based on our local experience, we recommend the use of dermis fat graft inserted within the scleral cavity. Epithelialization of the dermis is usually achieved in 4–6 weeks.

## 6. Future Perspectives

### 6.1. Avoiding Orbital Exenteration: Towards the Use of New Eye-Sparing Strategies

Most T1 and T2 CMs are managed conservatively with “no-touch” surgery followed by adjuvant treatments (see Section 4.2). As already mentioned, there is still no consensus on the most appropriate adjuvant treatment. The two main challenges for clinicians are

(i) the tumour recurrence;
(ii) and locally advanced tumours invading the fornix and/or the orbit.

In these highly complicated cases, OE has long been advocated to achieve definitive tumour control. As stated above, OE is a disfiguring surgical procedure, which is often refused by patients, its ability to reduce the metastatic spread having failed to be demonstrated. As for other locally advanced periocular malignancies, the last decade has been marked by the emergence of new eye-sparing strategies to avoid OE [98].

#### 6.1.1. Surgical Excision Followed by Proton Beam Therapy

Until recently, locally advanced CM was often considered an indication of OE. However, there is no clear consensus on the definition of the term “locally advanced”. It is usually acknowledged that ≥T2 tumours with a thickness ≥ 2mm, and with a fornix, caruncular or orbital involvement are associated with a poorer prognosis and an increased risk of metastatic spread [97,99]. To date, only a few authors have reported their experience with eye-sparing strategies in locally advanced CM [5,62]. In a preliminary study published in 2006, Wuestemeyer et al. treated 20 locally advanced CM patients with local excision followed by adjuvant PBRT [73]. Local recurrence and metastatic spread occurred in one third of the patients, in line with other series of cases with less advanced CM. Only 2 (10%) patients required OE. Interestingly, the best-corrected visual acuity (BCVA) remained stable over the study period in 60% of cases. Sicca syndrome was the most commonly reported complication. In 2019, the same team has reported additional results in 89 patients diagnosed with ≥T2 or multifocal T1 CM, treated conservatively with the same protocol [62]. Local recurrence and metastatic spread occurred in 33% and 16% of cases, respectively. Of the 89 patients, 18 (20%) underwent OE for inextirpable and/or recurrent tumour. The estimated 5-year cumulative likelihood of eye preservation was 69%. Overall, 11% and 8% of patients experienced secondary glaucoma and limbal stem cell deficiency, respectively.

**Figure 5:**
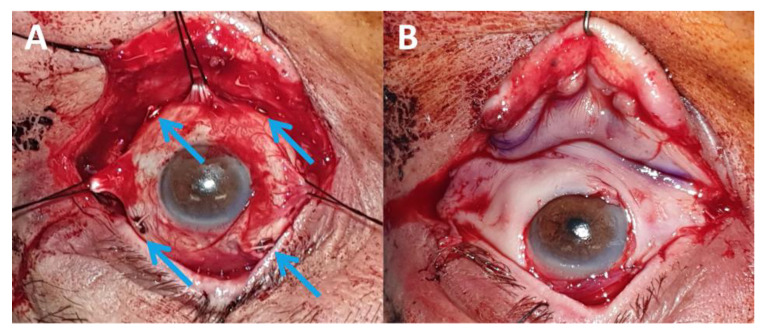
Removal of a conjunctival melanoma invading the inferior fornix. (**A**) Inferolateral cantholysis was performed to achieve adequate surgical exposure. The oculomotor muscles were dissected and hooked. Tumour removal involved 300° of the bulbar conjunctiva, the inferior conjunctival fornix and the posterior eyelid lamella (the anterior lamella and the tarsus were preserved). Four tantalum clips (blue arrows) were sutured to guide subsequent proton beam therapy; (**B**) Reconstruction of the entire defect with a buccal membrane graft with reconstruction of the lower fornix.

**Figure 6:**
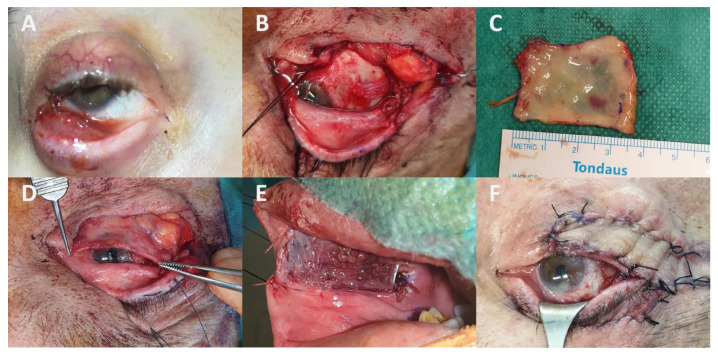
Wide full-thickness removal of a large conjunctival melanoma (CM). (**A**) CM invading the inferior fornix, the inferior bulbar conjunctiva and the inferior tarsal conjunctiva. (**B**) Tumour removal resulted in an 80% full-thickness lower eyelid defect and a 180° inferior bulbar conjunctiva defect; (**C**) A large buccal membrane graft was harvested; (**D**) The buccal membrane graft was positioned and allowed reconstructing the bulbar conjunctiva, the inferior fornix and the posterior lid lamella; (**E**) Direct closure was not possible. The defect was closed with a cryopreserved amniotic membrane graft sutured with interrupted 4/0 absorbable braided sutures + fibrin glue; (**F**) Anterior lamella was reconstructed with a dermatochalasis flap providing vascularization to the underlying oral membrane graft.

These 2 articles demonstrate the feasibility of conservative strategies in patients with locally advanced CM, as is the case for conjunctival carcinomas [100]. However, a major limitation of these articles is the lack of details regarding the surgical treatment prior to radiation therapy. In our opinion, achieving a R0 or R1 surgical excision is essential. A “no-touch” technique is used with at least 5-mm free macroscopic margins, as advocated for eyelid and orbital malignancies [84]. In our experience, removing ≥ T2 CM or CM invading the fornix is challenging and requires the following:

- a close cooperation between the ocular oncologist and the oculoplastic surgeon is essential;
- in the case of CM invading the fornix, the first surgical step is to perform inferior and/or superior cantholysis depending on tumour location to provide adequate surgical exposure (Figure 5);

- wide surgical removal with at least 5-mm free margins is recommended. The oculomotor muscles should be exposed and hooked if necessary. There is still no consensus on whether the anterior lid lamella should be preserved (Figure 5) or removed (Figure 6) in case of CM invading the fornix and the posterior lamella;

- the intraoperative placement of tantalum clips will help radiotherapists to deliver adjuvant PBRT more precisely, especially in the case of fornix involvement (Figure 3 and Figure 5).

Although several authors advocate the use of amniotic membrane grafts to reconstruct the fornixes in retracted sockets, we believe that buccal membrane grafts have several advantages over amniotic membranes grafts [101]. Buccal membrane grafts can be safely used in irradiated areas, are associated with a low rate of post-radiation retraction, can be used for a one-step reconstruction of the bulbar conjunctiva, the posterior lid lamella and the fornix (Figure 5B and Figure 6D), and have the potential to promote ocular surface healing by providing oral mucosa stem cells [102]. A large oral mucosa is often needed, making direct oral closure difficult. We agree with previous studies advocating the use of amniotic membrane grafts sutured in the oral cavity to reduce postoperative pain (Figure 6E) [103,104].

Although attractive, the enthusiasm aroused by conservative surgery followed by radiotherapy should be tempered because:

- data are currently scarce and are only based on retrospective studies;
- about one third of patients will experience tumour recurrence requiring revision surgeries +/− radiotherapy;
- complications are common (sicca syndrome, cataract, secondary glaucoma, corneal ulcer) and may impair patient’s quality of life [62,73];
- although the globe is preserved, several patients will progressively lose their vision (secondary glaucoma, limbal cell deficiency); therefore, a distinction should be made between eye-sparing and sight-sparing strategies [98];
- about 20% of patients will ultimately undergo secondary OE with possible delays in socket wound healing and orbital implant osseointegration failure due to previous radiotherapy [62,105];
- not all CMs are eligible for conservative surgery.

**Figure 7:**
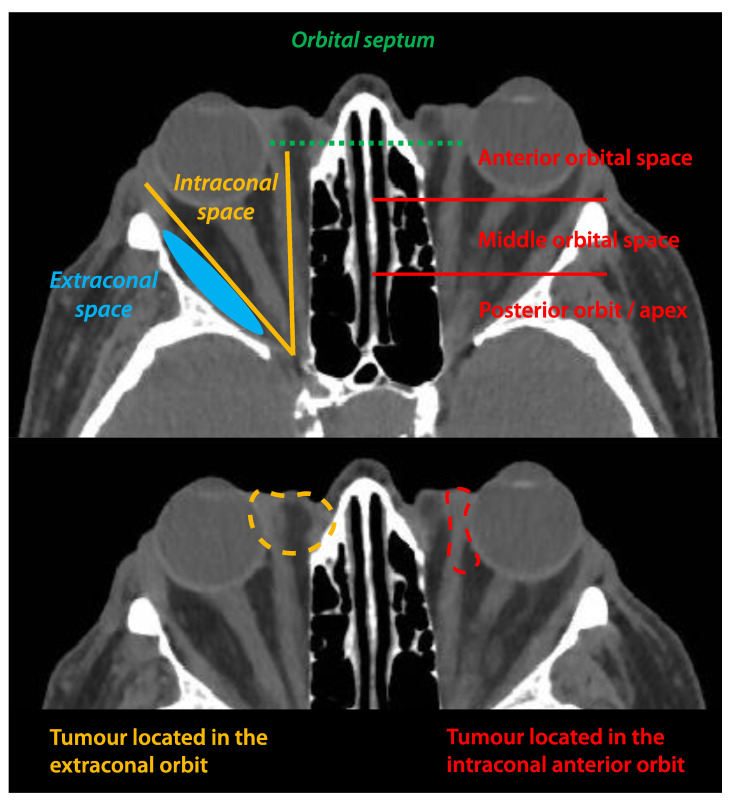
Schematic representation of the different surgical orbital spaces. Conjunctival melanomas located in the intraconal space are not accessible to conservative surgery.

An intraorbital involvement is defined by a tumour spread located behind the orbital septum (Figure 7). Compared to locally advanced basal cell carcinoma (BCC), only malignancies invading the anterior and extraconal orbital spaces can be treated conservatively (Figure 7) [106,107]. Removal of more posterior and/or intraconal malignancies is associated with major surgical difficulties and subsequent iatrogenic damage [96]. In addition, the data on BCC cannot be strictly extrapolated to CM because adjuvant radiotherapy is rarely required in BCC [104,105]. In the case of CM invading the anterior orbit, adjuvant radiotherapy would be indicated despite complete surgical excision, due to its invasive and infiltrative nature. To date, data on conservative surgeries followed by adjuvant radiotherapy for CM invading the orbit are lacking.

#### 6.1.2. Development of Targeted Therapies and Immunotherapies for Implementing New “Medical” Eye-Sparing Strategies

The last decade has been marked by a better understanding of the genetics of CM. An overactivation of the MAPK pathway and, to a lesser extent, of the PI3K-AKT pathway, has been noted [12]. As with cutaneous melanoma, this has led to consider CM as a malignancy that is “targetable” by MAPK inhibitors. BRAF mutation screening has now become a routine practice, with particular attention to BRAF non-V600E mutations [1,108]. At the time this review was written, only a few case series, including fewer than 10 metastatic CM patients treated with BRAF inhibitors alone or in combination with MEK inhibitors, were published. A complete or partial response was achieved in about half of the cases [109]. As for cutaneous melanoma, targeted therapies combining BRAF and MEK inhibitors will probably be considered as the treatment of choice [110].

Immunotherapy has also gained in interest in locally advanced or metastatic CM. Anti-CTLA-4 and anti-PD-1 immunotherapies have been approved by the FDA in 2011 and 2014, respectively, for the treatment of metastatic cutaneous melanoma. Since then, numerous studies have been published supporting the fact that PD-1 inhibitors given alone were more effective than CTLA-4 inhibitors given alone, and that a combination of both immunotherapies could be more effective than monotherapy without increasing the adverse effects [111]. Several studies have reported an overexpression of PD-L1 in CM specimens, but it should be noted that immune cells within the TME showed a stronger staining than the tumour cells [12,43]. This could explain why the presence of peritumoral immune infiltrates has been associated with a better overall survival [12]. To date, fewer than 20 patients have been treated with PD-1 inhibitors alone, CTLA-4 inhibitors alone, or a combination of both. High rates of complete and partial responses have been reported [112,113]. Interestingly, favourable outcomes have been reported, even for patients with tumours that did not (over)express PD-L1. Contrary to the assessment of the *BRAF* mutational status, the assessment of PD-L1 expression in tumour samples is not recommended in daily practice [12].

Compared to more traditional strategies based on surgery and adjuvant brachytherapy or PBRT, these new medical treatments virtually allow treating all CMs, regardless of their degree of orbital invasion (Figure 7).

Despite favourable outcomes, targeted therapies and immunotherapies raise many questions and should be used with caution. For example, the following should be noted:

- the current literature is scarce and may be affected by a publication bias towards treatment. There is currently no ongoing clinical trial investigating MAPK inhibitors and immune checkpoint inhibitors in CM;
- not all CMs are targetable by BRAF and MEK inhibitors. Preliminary data indicate that NRAS-mutated CMs are relatively resistant to targeted therapies [114]. Therefore, only CM carrying a BRAF mutation can be treated with MAPK inhibitors;
- targeted therapies are especially indicated in more advanced cases. However, BRAF mutations are thought to occur early in CM oncogenesis with a higher prevalence in T1 tumours compared to later stages [21,108];
- targeted therapy and immunotherapy adverse effects are frequently reported (in about 90% of patients treated with MAPK inhibitors) and can lead to treatment discontinuation;
- as with cutaneous melanoma, initial favourable responses have been reported followed by secondary relapses within a year, especially when BRAF or MEK inhibitors were given alone. Secondary resistance mechanisms are not yet elucidated and studies with a longer follow-up are needed to better assess their incidence in CM. Further studies assessing the rate of secondary resistance to combined BRAF and MEK inhibitors are also needed;
- treatment duration is not consensual;
- cost-effectiveness analyses are currently lacking;
- the treatment protocol remains to be established.

There is still no consensus on targeted therapy and immunotherapy prescriptions. Several authors have only treated metastatic (lymphatic and/or hematogenous) CMs, whereas others have treated locally advanced (e.g., ≥T2) CMs without metastatic spread [112]. When targeted therapies or immunotherapies are prescribed to preserve the eyeball, should treatment be considered curative (i.e., until complete response is achieved) or neoadjuvant (i.e., until tumour growth becomes accessible to less invasive surgery and radiotherapy)?

### 6.2. Treatment of Metastatic Conjunctival Melanoma

There is currently no standard therapy regimen for the treatment of metastatic CM. Targeted therapies and immunotherapies have shown promising results in metastatic CM (Table 2). To date, consensus conference recommendations on the management of metastatic melanoma are available and offer different options to be propose to patients. but no specific recommendations have been proposed for metastatic CM [115].

Given the phenotypic and molecular similarities between CM and cutaneous and mucosal melanomas:

Systemic immunotherapies widely used in cutaneous melanoma for several years could also be beneficial to patients with metastatic CM [44,112,113,116,117,118];

Treatments targeting KIT and BRAF that are standard in cutaneous melanoma are also likely to be effective in CM [118,119,120,121,122,123,124].

These considerations and the published case reports support the inclusion of CM (a rare disease) in skin melanoma trials.

**Table 2:** Previous reports of treatment in patients with metastatic disease.

Study	Patient Gender, Age	Disease Sites	Prior Treatments	Mutational Status	Drug, Dosage, Duration (months)	Follow-Up after Treatment (Months)	Clinical Outcome ‡	Adverse Events (Grade) †
**Targeted therapy**								
Kiyohara et al., 2020 [119]	M, 72	lymph node	lymph node dissection (parotidectomy)	*BRAF^V600E^* mutation	dabrafenib + trametinib, NR (6)	0 (under treatment)	CR	NR
Rossi et al., 2019 [120]	M, 70	lymph node	lymph node dissection (parotidectomy)	*BRAF^V600E^* mutation	dabrafenib + trametinib, 150 mg for 2 d + 2 mg for 1 d (8)	0 (under treatment)	PR	fever hypertransaminasemia (1)
Pinto Torres et al., 2017 [118]	F, 56	hematogenous (orophanryngeal wall)	surgery EBRT (20 Gy/5 fr)	*BRAF^V600^* mutation	vemurafenib, 960 mg for 2d then 480 mg for 2 d due to AE (34)	6	CR developed breast cancer	skin rash (1) arthralgia (2) diarrhoea (2)
Maleka et al., 2016 [121]	F, 53	hematogenous (orbit, brain, lung)	enucleation temozolomide (5 m) AdCD40L + cyclophosphamide whole brain EBRT (20 Gy/5 fr)	*BRAF^V600E^* mutation	vemurafenib, 960 mg for 2 d then 240 mg for 2 d due to AE (4)	5	Progression (for orbital location, PR for other locations) Death	skin rash (2)
Griewank et al., 2013 [122]	M, 43	hematogenous (intramuscular, lungs, brain)	dacarbazine	*BRAF^V600^* mutation	dabrafenib, NR (6)	NR	Progression (initial PR)	NR
Weber et al., 2013 [123]	M, 45	hematogenous (subcutaneous, lungs, bone)	none	*BRAF^V600E^* mutation	vemurafenib, 960 mg for 2 d (3)	NR	Progression	NR
**Immunotherapy**								
Hong et al., 2021 [116]	M, 66	hematogenous (lungs, liver)	none	NR	ipilimumab + nivolumab, 3 mg/kg + 1 mg/kg (6 cycles)	4	NR (response without detail)	hypopituitarism (2)
Chang et al., 2019 [44]	F, 60	hematogenous (liver)	none	*NRAS* mutation	1: ipilimumab + nivolumab, 3 mg/kg + 1 mg/kg (2 cycles) 2: ipilimumab, 240 mg (2 cycles) then 480 mg (1 cycle) 3: pembrolizumab, 200 mg (9 cycles)	24	NR (response without detail)	1: hepatitis (3) 2: allergy (NR) 3: NR
Finger and Pavlik, 2019 [113]	F, 76	lymph node	lymph node dissection (parotidectomy) + EBRT (cervical + mediastinal)	*NRAS^Q61R^* mutation	1: ipilimumab, 3 mg/kg (4 cycles) 2: surgery + EBRT + ipilimumab, 3 mg/kg (4 cycles) 3: surgery + EBRT (50 Gy/20 fr) + pembrolizumab, 2 mg/kg (14 cycles)	24	CR	NR
	F, 72	hematogenous (lungs, liver, bone, subcutaneous, node)	none	*BRAF^V600K^* mutation	ipilimumab + nivolumab, 3 mg/kg + 1 mg/kg (3 cycles)	36	CR	liver toxicity (2) colitis (3) pneumonitis (2)
Chaves et al., 2018 [117]	M, 72	lymph node	lymph node dissection (parotidectomy)	NR	ipilimumab, 3 mg/kg (4 cycles)	16	CR	fatigue (2)
Sagiv et al., 2018 [112]	F, 58	hematogenous (lungs, liver)	none	NR	nivolumab, 3 mg/kg (6 cycles)	9	CR	biological hepatic failure (3)
	F, 28	hematogenous (breast, lungs, clavicle, thigh)	none	NR	nivolumab, 3 mg/kg (7 cycles)	36	CR	NR
	F, 47	hematogenous (lungs)	none	NR	nivolumab, 3 mg/kg (10 cycles)	7	CR	colitis (3) diarrhoea (3)
	M, 74	hematogenous (lungs)	none	NR	nivolumab, 3 mg/kg (22 cycles)	1	PR	colitis (3)
Pinto Torres et al., 2017 [118]	M, 51	lymph node	cervical lymphadenectomy antiviral therapy for HIV	no *BRAF* mutation	pembrolizumab, 2 mg/kg (12 cycles)	0 (under treatment)	CR	NR
**Combination therapy (immunotherapy and targeted therapy)**								
Kiyohara et al., 2020 [119]	M, 71	lumbar vertebra	Enucleation + vemurafenib	*BRAF^V600E^* mutation	1: dabrafenib + trametinib, NR (6) 2: EBRT + nivolumab, NR (NR) 3: dabrafenib + trametinib, NR (NR)	NR	Death (24 months after initial treatment)	1: skin rash (2) 2–3: NR
Sagiv et al., 2018 [112]	F, 68	hematogenous (lungs)	none	no *BRAF^V600E^* mutation	1: pembrolizumab, 2 m/kg (13 cycles) 2: ipilimumab + dacarbazine, 3 mg/kg + 800–1000 mg/m^2^ (2 cycles)	0	PR	1: NR 2: hepatic failure (4)
Dagi Glass et al., 2017 [124]	F, 61	lymph node	lymph node dissection (parotidectomy) + cervical lymphadenectomy	*BRAF^V600E^* mutation	1: dabrafenib + trametinib, NR (1.5) 2: vemurafenib, NR (3.5) 3: pembrolizumab, NR (2) 4: vemurafenib, NR (4) 5: vemurafenib + cobimetinib, NR (24)	0 (under treatment)	CR	1: nausea (3) 2–5: NR

NR: not reported; CR: complete response; PR: partial response; HIV: human immunodeficiency virus; EBRT: electron beam radiotherapy; † according to the Common Terminology Criteria for Adverse Events (5th edition); ‡ according to response evaluation criteria in solid tumours (RECIST) 1.1 (no information available for immunotherapy response evaluation criteria in solid tumours).

### 6.3. Perspectives: Towards Personalized Treatment

**Figure 8:**
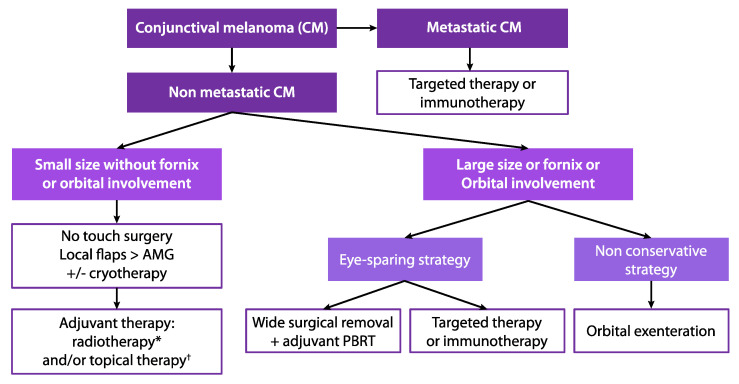
Current management proposal for early-stage and advanced conjunctival melanoma. *proton beam radiotherapy or brachytherapy in case of invasive melanoma; † topical chemotherapy (mitomycin C 0.04%) or topical immunotherapy (interferon alpha 2b) in case of melanoma in situ or primary acquired melanosis with moderate to severe atypia.

Given CM’s scarcity, the lack of consensus on the best treatment to reduce local recurrences, the development of eye-sparing strategies and new targeted therapies, CM has become an especially complex malignancy, requiring multidisciplinary management (Figure 8). The usefulness of SLN biopsies is not consensual, but their analysis could be relevant in locally advanced (≥T2) CMs [12,54]. Recurrently, the management of locally advanced and disseminated CMs should be discussed in specialized ocular oncology centres with a close cooperation between dermatologists, oncologists, pathologists, and radiotherapists. A systematic checklist, including the tumour stage (TNM), the tumour recurrence status, the visual acuity of the affected and contralateral eyes, the BRAF mutational status, and the positivity of the SLN biopsy, as well as the overall health status of the patient, might help clinicians offer more personalized treatment in the future. Moreover, new single-cell analysis techniques might allow a better understanding of CM biology and the identification of new therapeutic targets, as was recently shown for uveal melanoma [125,126,127,128]. Such analyses in CM could allow a combination of existing or new treatments where several cellular subpopulations are identified.

## 7. Conclusions

Despite its small size, CM remains a highly challenging periocular malignancy. Despite complete local excision, about one third and one quarter of patients will experience local recurrence and systemic spread, respectively. The last decade has been marked by the desire to alleviate the need for invasive and disfiguring OE. Therefore, new eye-sparing strategies based on wide surgical resections, followed by customized PBRT, have emerged as viable procedures. More importantly, new genetic insights have allowed making CM a targetable malignancy. MAPK inhibitors and immunotherapies have shown favourable and promising results in terms of eye preservation and metastatic spread. CM is currently the most “multidisciplinary” ophthalmic malignancy, and requires a close cooperation between the ocular oncologist, the oculoplastic surgeon, the onco-dermatologist, the pathologist, the oncologist, and the radiotherapist.

## Figures and Tables

**Table 1:** Case series of more than 10 patients describing the use of radiotherapy for treating conjunctival melanoma.

Study	Cases	Follow-Up in Months (Mean (Range))	cTNM (*n* (%))	Origin of the Tumour (*n* (%))	Thickness (mm) (Mean (Range))	Adjuvant Therapy after Primary Surgery (with or without Cryotherapy) (*n* (%))	Target of Initial Treatment	5-Year Local Recurrence Rates, %
Pacheco et al., 2021 [7]	629	58 (<1–336)	*n* = 425 (100) T1 = 266 (63) T2 = 75 (18) T3 = 80 (20)	*n* = 629 (100) PAM = 476 (76) Naevus = 59 (9) De novo = 94 (15)	*n* = 476 2.7 (0.2–20.0)	*n* = 30 (5) ^†^ - Topical CT (MMC) = 19 (3) - Topical IT (IFN-a2b) = 1 (<1) - RT = 10 (2) Plaque = 6 (1) EBRT = 4 (1)	NR	Overall = NR PAM = 40 Naevus = 28 De novo = 42
Brouwer et al., 2021 [52]	58	97.3 (9.3–229)	*n* = 58 (100) T1 = 57 (98) T2 = 1 (2)	*n* = 58 (100) PAM = 52 (90)	*n* = 58 0.9 (NR)	*n* = 58 (100) - Topical CT (MMC) = 15 (26) - RT (plaque) = 58 (100)	- On site = 20 (34) - Other site = 38 (66)	Overall = 21
Jain et al., 2020 [66]	288	52.8 (1–171)	*n* = 288 (100) T1 = 218 (76) T2 = 34 (12) T3 = 15 (5) Tx = 21 (7)	NR	*n* = 271 1.9 (0.2–16)	*n* = 199 (69) - Topical CT = 109 (38) MMC = 107 (37) 5-FU = 2 (<1) - Topical IT (IFN-a2b) = 20 (7) - RT = 106 (37) Plaque = 55 (19) EBRT = 15 (5) PBRT = 36 (14)	NR	Overall = 19
Thariat et al., 2019 [5]	92	56.4 (NR)	*n* = 88 (100) T1 = 63 (72) T2 = 13 (15) T3 = 12 (13)	*n* = 92 (100) PAM = 60 (65)	*n* = 92 2.5 (1.0–4.0)	*n* = 92 (100) - Topical CT (MMC) = 22 (24) - RT (PBRT) = 92 (100)	- On site = 42 (46) - Other site = 50 (54)	Overall = 33
Scholz et al., 2019 [62]	89	50.4 (1–260)	*n* = 89 (100) T1c/d= 5 (6) T2 = 49 (55) T3 = 35 (39)	*n* = 89 (100) PAM = 53 (60)	NR	*n* = 89 (100) - Topical CT = 22 (25) - RT = 89 (100) Plaque = 12 (8) PBRT = 89 (100) - Combination ^b^ = 4 (5)	NR	NR
Brouwer et al., 2018 [69]	70	70.2 (3–172)	*n* = 70 (100) T1 = 54 (77) T2 = 16 (23)	*n* = 70 (100) PAM = 65 (93)	*n* = 54 2.3 (NR)	*n* = 39 (56) - Topical CT (MMC) = 1 (1) - RT = 38 (54) Plaque = 34 (49) EBRT = 4 (6)	- On site = 48 (69) - Other site = 22 (31)	Overall = 29
Larsen et al., 2015 [70]	132	73.2 (4–528)	*n* = 47 (100) T1 = 32 (68) T2 = 11 (23) T3 = 4 (9)	*n* = 129 (100) PAM = 80 (62) Naevus = 33 (26) Naevus + PAM = 2 (2) De novo = 14 (11)	NR	*n* = 18 (14) ^‡^ - Topical CT = 3 (2) - RT (plaque) = 15 (12)	NR	NR
Cohen et al., 2013 [71]	20	59 (8–152)	*n* = 20 (100) T1 = 20 (100)	PAM = 15 (75) De novo = 5 (25)	*n* = 17 2.1 (0.6–6)	*n* = 20 (100) - Topical CT (MMC) = 1 (5) - RT (plaque) = 20 (100)	NR	Overall = 18
Karim and Conway, 2011 [72]	19	43.1 (30–54)	*n* = 19 (100) T1 = 19 (100)	PAM = 19 (100)	*n* = 19 0.7 (median) (0.2–1.6)	*n* = 19 (100) - RT (plaque) = 19 (100)	NR	0
Savar et al., 2011 [53]	26	32 (2.4–84)	*n* = 26 (100) T1 = 9 (35) T2 = 10 (38) T3 = 7 (27)	NR	*n* = 23 2.7 (0.23–12)	*n* = 9 (35) - Topical CT (MMC) = 5 (19) - Topical IT (IFN-a2b) = 1 (4) - RT (EBRT) = 3 (12)	- On site = 20 (69) - Other site = 6 (21)	Overall = 9
Damato and Coupland, 2009 [51]	76	52.8 (median) for patients initially treated on site 38.4 (median) (8–167) for referred patients	NR ^a^	NR	NR	NR	- On site = 40 (53) - Other site = 36 (47)	NR
Wuestemeyer et al., 2006 [73]	20	38.1 (NR)	*n* = 20 (100) T1 = 2 (10) T2 = 14 (70) T3 = 4 (20)	PAM = 2 (10) De novo = 2 (10) NR = 16 (80) (recurrence)	NR	*n* = 20 (100) - Topical CT = 2 (10) - RT = 4 (20) Plaque = 3 (15) EBRT = 1 (5) PBRT = 20 (100) - Combination ^b^ = 2 (10)	NR	Overall = 40 (but mortality at 3 years)
Missotten et al., 2005 [6]	194	81.6 (1–618)	NR ^a^	*n* = 194 (100) PAM = 111 (57) Naevus = 3 (2) Naevus + PAM = 9 (5) De novo = 50 (26) Inconclusive = 21 (11)	*n* = 152 2.07 (NR)	*n* = 35 (18) - Topical CT = 4 (2) - RT = 31 (16) Plaque = 20 (10) EBRT = 11 (6)	NR	Overall = 39
Tuomaala et al., 2002 [40]	85	75.6 (3–396)	NR ^a^	*n* = 77 (100) PAM = 53 (69) Naevus = 23 (30) NR = 1 (1)	*n* = 72 1.3 (0.2–8.8)	*n* = 6 (7) - Topical CT = 5 (6)	NR	Overall = 36
Werschnik and Lommatzsch, 2002 [74]	85	165.6 (NR)	*n* = 85 (100) T1 = 48 (56) T2 = 37 (44)	*n* = 85 (100) PAM = 22 (26) Naevus = 29 (34) De novo = 34 (40)	NR	*n* = 38 (45) ^c^ - RT (plaque) = 38 (45)	NR	Overall = 40
Anastassiou et al., 2002 [75]	69	67 (median) (15–360)	NR ^a^	*n* = 69 (100) PAM = 29 (42) Naevus = 27 (39) De novo = 11 (16) Inconclusive = 2 (3)	NR	*n* = 40 (58) - Topical CT = 3 (4) - RT = 34 (49) Plaque = 20 (29) EBRT = 10 (14) PBRT = 2 (3) - Combination ^b^ = 1 (1)	NR	NR

NR: not reported; CT: chemotherapy; IT: immunotherapy; RT: radiotherapy; IFN-α2b: interferon α 2b; MMC: Mitomycin C; 5-FU: 5-Fluorouracil; EBRT: electron beam radiotherapy; PBRT: proton beam radiotherapy; PAM: primary acquired melanosis. ^a^ NR for the 8th classification; ^b^ Combination of chemotherapy/brachytherapy or chemotherapy/external beam radiotherapy; ^c^ 7 patients (not included in the 38) were irradiated without surgery; ^†^ data available for 609 patients; ^‡^ data available for 129 patients.
